# A narrative study of mental health recovery: exploring unique, open-ended and collective processes

**DOI:** 10.1080/17482631.2020.1747252

**Published:** 2020-04-04

**Authors:** Nina Petersen Reed, Staffan Josephsson, Sissel Alsaker

**Affiliations:** a Department of Mental Health, Norwegian University of Science and Technology, Trondheim, Norway; b Department of Neurobiology, Care Sciences, and Society, Karolinska Institutet, Stockholm, Sweden

**Keywords:** Ethnography, everyday activities, meaning making, mental health, narrative, participant observation, recovery

## Abstract

**Purpose**: Guided by narrative theory and by use of a narrative-in-action approach, the aim of this study was to explore how mental health recovery unfolds through individuals’ engagement in everyday activities.

**Method**: Data were created through participant observations with four individuals while doing everyday activities, and analysed through a narrative, interpretive approach.

**Findings**: The findings show how mental health recovery involves unique and open-ended processes of narrative meaning-making, which unfold through an interplay between everyday activities, places and persons.

**Discussion**: Based on these findings, we discuss how we may understand and support mental health recovery as collective processes.

## Introduction

Everyday activities are an important focus for recovery-oriented research and practice. Doing everyday activities is our way of structuring and creating meaning in our lives (Hammell, 2004; Wilcock, 1999), and holds potential for healing and transformation (Mattingly, 1998; Townsend, 1997). The transformative potential of everyday activities has been explored in research on mental health recovery, suggesting that recovery progresses through activities and describing recovery as an occupational journey embedded in everyday life contexts (Borg & Davidson, 2008; Davidson et al., 2006; Doroud et al., 2015; Kelly et al., 2010; Sutton et al., 2012). However, mental illness may bring about major interruptions to individuals’ everyday lives and social relations (Zolnierek, 2011), including not being able to do the everyday activities they have previously engaged in (Alsaker & Ulfseth, 2017; Baker & Procter, 2014), and sometimes needing support from professionals and others to carry out activities (Yilmaz et al., 2009).

Acknowledging the importance of everyday activities in recovery, several authors call for more in-depth, processual and contextual knowledge of how processes of recovery unfold through everyday activities (Doroud et al., 2015; Duff, 2016; Ellison et al., 2018; Price-Robertson et al., 2017; Topor et al., 2011). Research shows how recovery is complex and contextually dependent, involving multiple processes of regaining connectedness, hope and optimism about the future, identity, meaning in life, and empowerment (Le Boutillier et al., 2011; Slade et al., 2012), as well as dealing with difficulties (Stuart et al., 2017). Social factors and relationships (Tew et al., 2012; Topor et al., 2006), as well as places (Duff, 2012; Myers, 2016) are found to be important dimensions in recovery. As research suggests a multifaceted understanding of recovery, there has been some critique on research and services that focus primarily on the individual, with social and contextual factors serving only a secondary role (Kogstad et al., 2011; Price-Robertson et al., 2017).

Narrative theory may help understand recovery as processes unfolding through the activities and experiences of everyday life. In a narrative, several elements such as persons, activities, events and contexts are drawn together into a coherent story which conveys a possible plot or meaning of human activity (Polkinghorne, 1995). A narrative plot is a thematic thread related to important issues in individuals’ lives which may clarify the meaning of separate actions or events, through connecting them in the narrative as a whole (Bruner, 1990; Polkinghorne, 1995). Narratives can be both told and enacted, and everyday activities may be understood as part of ongoing enacted narratives with open endings, entailing opportunities of healing and transformation (Mattingly, 1998). Further, enacted narrative meaning making is described as an ongoing and creative process of creating coherence through trying out in thoughts and actions plots that connect past and present activities and experiences to our ideas and wishes of future scenarios (Alsaker & Josephsson, 2010; Josephsson et al., 2006; Ricoeur, 1983). Thus, viewing recovery as processes of narrative meaning-making inspires exploration of how persons, everyday activities, experiences, hope and visions for the future, places and contexts may be understood in relation to each other and form narratives of recovery.

A narrative understanding of recovery also sheds light on the relational nature of these processes. Bruner (1990, p. 73) writes that inherent in processes of narrative meaning-making is a sensitivity towards others, a “social meaning readiness”. If others cannot make sense of our narratives, they may fall apart, requiring us to negotiate and adjust them (Bruner, 1990; McAdams, 2006). Demonstrating the relationality of narratives of recovery, several studies show how everyday activities that put us in touch with others are particularly valuable to create meaning. When doing activities together, the persons involved try out possible plots in collaboration, seeking to create narratives that make meaning to everyone involved (Lindström et al., 2013; Ørjasæter et al., 2017; Reed et al., 2018; Ulfseth et al., 2015, 2016).

Lastly, a narrative understanding embraces the complexities of recovery as un-linear processes. Recovery presents those involved, both the person with mental health challenges, family, friends, professionals and/or others, with hurdles such as having to make difficult choices, having to negotiate and try out several courses of action, experiencing disruptive symptoms, stigma and lack of support and resources, as well as losses, setbacks and failed attempts (P. Deegan, 1988; Reed et al., 2017; Zolnierek, 2011). P. E. Deegan (2002) describes recovery as processes of creating transformation narratives, discovering both one’s limits and possibilities. Roe and Davidson (2005) underline how mental illness may result in disrupted life narratives and understand recovery as an effort of re-creating coherence and meaning by “gathering up the pieces” of one’s previous life and putting them together again through trying out, improvising, and negotiating. Correspondingly, narrative plots are not associated with straight and smooth threads, but rather messy threads with occasional knots, frizzles and loose ends, causing tension and suspense (Mattingly, 1998). Hence, understanding recovery as narrative processes may help shed light on its complexity and underline its openness to negotiations and rearrangements, instead of viewing it as processes with well-defined dimensions and endings.

Based on the literature reviewed here we understand that processes of mental health recovery are multifaceted, relational and open-ended. We propose that viewing recovery as a process of narrative meaning-making may help understand how persons, everyday activities and contexts are connected in these processes, explore the importance of relationships, and keep in mind the complexities and open-endedness of these processes. Answering to the call for more processual and contextual knowledge about mental health recovery we therefore build on a narrative understanding of recovery in this study, and our aim is to explore how mental health recovery unfolds through individuals’ engagement in everyday activities.

## Method

Aligning with our narrative understanding of recovery, we chose a narrative-in-action approach for this study, building on narrative theory and methodology (Bruner, 1990; Mattingly, 1998; Ricoeur, 1983, 1986), and the work of Alsaker and Josephsson, and their colleagues (Alsaker & Josephsson, 2010; Alsaker et al., 2009; Josephsson & Alsaker, 2014). This qualitative, ethnographic approach focuses on exploring how individuals make meaning through what they do, when, where and with whom.

### Recruitment of participants

The first author contacted the three community mental health centres in an urban municipality in Norway. These centres are run by the municipality and serve as local meeting places for individuals with mental health challenges. Here people can stop by to spend time with others and engage in activities organized by both service users and staff, such as meals, art-groups and physical activity. The centres invited the first author to inform about the study and call for participants at their house meetings, as well as through written information on their notice board. We called for participants who experience mental health challenges affecting their daily lives, who were currently living at home in the community, and who were interested in creating knowledge about mental health and everyday living. Individuals interested in participating in the study were encouraged to contact the first author, either directly or through the staff at the centre. Two men and two women, all in their 40 s or 50 s, contacted the first author willing to participate in the study. Before starting data generation, the participants signed written consent forms.

### Data generation

The data in this study was co-created by the participants and the first author, following recommended guidelines provided in the literature on ethnography (Fangen, 2004; Hammersley & Atkinson, 2007). The first author met with each participant 7–8 times, over a period of 6–8 months. Each meeting lasted from 2 to 4 hours while doing everyday activities suggested by the participants. Several of these meetings took place at the community mental health centres, other meetings took place while doing activities such as working out at the gym, hiking in the woods, or drinking coffee and talking at home. Before and after each meeting, the first author wrote field notes describing her preparations and preunderstandings, the contexts, events and conversations taking place during the meeting, and her analytical reflections after the meeting. These texts, in total about 49,500 words, formed the data material.

### Data analysis

We analysed the data using a narrative, phenomenological-hermeneutic approach (Josephsson & Alsaker, 2014; Polkinghorne, 1995). In narrative analysis, the researchers seek to develop or discover plots that displays a linkage between different data elements and how they together make meaning as contributors to goals or purposes (Polkinghorne, 1995, p. 15). We used the narrative understanding of recovery described in our introduction as an analytical framework, guiding our focus and interpretations.

During the process of analysis, all three authors met on several occasions to discuss preliminary findings and interpretations. To begin the analysis, the first and third author read the field notes to get an overview of the data material. The first author then re-read the field notes several times, searching for events that raised curiosity or questions related to the aim of the study. Such puzzling parts of the data material may function as “significant events” (Mattingly, 1998), uncovering possible plots (Josephsson & Alsaker, 2014). As an example, Sandra’s story about how she suddenly overcame her anxiety and became active at the community mental health centre puzzled us. How and why did this come about? We understood this as a possible significant event and made it the starting point of our analysis.

After having identified possible significant events, the first author read the field notes again, searching for other parts of the data material that seemed connected to them. Following up on our example, we further explored and interpreted Sandra’s story about how she became active at the centre by trying out connections with her current situation of being a user-representative and mother, as well as data about her past. Hence, the process of analysis followed the principles of a hermeneutic circle (Gadamer, 1988), expanding our understanding by moving between parts and whole in the data material. Our interpretations were further developed through drawing on narrative theory as mentioned above, as well as relevant research literature about recovery and narrative meaning-making, fulfiling a double hermeneutic spiral of interpretation (Giddens, 1993).

As next step in our analysis, we constructed narratives from these events by pulling them together into stories with a possible emergent plot (Josephsson & Alsaker, 2014). This helped us further develop and communicate the findings and interpretations that we present in this article. The first author then met with each of the participants to present, explain and discuss our findings and interpretations of the data created with them. All four participants stated that the focus of our analysis is relevant and important for them and that they could recognize our interpretations. This improved the validity of our findings. However, it is important to note that the narratives presented here are mainly the authors’. Further, this study explores recovery as it unfolds, hence these are not narratives representing completed processes of recovery. Rather they must be viewed as possible interpretations related to our study aim, grounded in theory and research.

### Ethical considerations

The study was approved by the regional committee for medical and health research ethics (approval number: 2013/2410/REK midt), as well as the director of health in the municipality of study. We changed names and details to ensure participant confidentiality. A narrative case study with data from this project is published elsewhere, exploring an everyday event of recovery (Reed et al., 2018).

When generating data through participant observations over time and in everyday situations, we found it important to create open and trusting relationships, but at the same time keeping professional boundaries (Lawlor & Mattingly, 2001). The first author therefore repeated and confirmed the nature and temporality of the relationships with the participants throughout the meetings, keeping the relationships professional. In addition, through working several years in community mental health services the first author has experience in building trusting relationships, communicating and supporting persons with mental health challenges. These experiences assisted the first author’s sensitivity and reflexivity regarding the participant–researcher relationship, while being careful not assuming the role of a mental health professional, or “helper”, in the conversations and activities shared with the participants.

## Findings

We here present our findings, showing how four individuals pursue recovery in their own unique ways. In our interpretation, these individuals seek to create meaning from and through everyday experiences and activities, by using their narrative capacities to try out possible plots through thoughts, everyday activities, and communication. Evident in our findings is how these ongoing processes of recovery are ambiguous and open-ended, as well as how everyday activities involve interplays between places and persons that are essential for these individuals’ recovery.

First, we present Brad and our analysis of how his process of recovery seems dependent on organizing and doing everyday activities with others.

### Driving thoughts into action through collective meaning-making

Brad is a man in his mid-fifties. He previously lived with his wife and children, worked full-time as an academic, and engaged in volunteer community work. However, some years ago Brad experienced severe mental illness. He now lives alone, receives disability pension and spends several days a week at the community mental health centre. Although Brad is an active contributor in both initiating, organizing and doing activities at the community mental health centre, he also seems to be in search of new possibilities:

One day while visiting Brad in his apartment, I complemented his view towards a walkway by the river. Brad laughingly replied, “Yes, here I sit in my sofa and watch people rushing by … ” Brad confessed that he sometimes feels bored, and that he wishes to become more active. Brad mentioned several activities he would have liked to do, like photographing, political discussion groups and cooking classes. He wished that the community mental health center would offer some of these activities. He also said that he would like to work again, but soon dismissed this idea, saying: “But I would never be able to acquire paid work of my liking, and then I will not feel motivated”.

We here understand that Brad is in a process of imagining activities to engage in, that would contribute to becoming more active. Several of the activities Brad mentions are activities he used to engage in before he became ill. Looking back at activities he has previously enjoyed and mastered, he imagines doing some of them again. However, we understand that Brad adjusts his images of future scenarios based on perceived limits and possibilities in his current situation, and consequently chooses not to pursue work as a possibility.

Brad communicates that being active and social are important issues for him, and we therefore wonder if these issues may be possible plots that can connect his active past, with his present activities and images for the future. However, because of the disruptions caused by mental health problems, Brad is unable to engage in the same everyday activities and social networks as before. We understand these disruptions as knots he needs to disentangle, causing tension and suspense, and requiring him to imagine and test new possibilities of being and becoming active. Brad currently seems to rely on the community mental health centre as an arena to do this:

One day while working out together, Brad told me that he had previously enjoyed attending yoga-classes at the community mental health center. Brad said that he wished to invite a yoga-instructor to the center again. However, seeming discouraged Brad underlined that he is not able to make such initiatives entirely on his own. He told me that he would need help and motivational support from the staff, but they had not provided this … Although Brad was unhappy about this, he sighed and said, “Without the center I do not know what I would do”.

Brad here describes that he needs someone to share his idea with, who also takes initiatives to organize and engage in yoga together with him. We understand that Brad recognizes the community mental health centre as a safe place where such interplays may come about. However, Brad’s initiative to engage the staff in yoga was unsuccessful, seemingly leaving him unable to drive this idea into action. Thus, Brad’s process of trying out yoga is currently in suspense, awaiting the contributions of the staff at the centre to tie up this loose end. We understand that although Brad imagines possibilities for becoming more active, he needs the shared enthusiasm and active engagement of others to drive his ideas into action. Thus, Brad’s process of meaning-making seems to unfold not simply through imagining and trying out activities, but through engaging in activities that involve interplay with others where they try out ideas and activities together, and thereby create meaning collectively.

In the next section, we present Carl, and our analysis of how recovery requires engagement in activities, places and interactions significant to his unique process of narrative meaning-making.

### Pursuing work as an architect—narrative meaning-making in suspense

Carl is a man in his late forties. Soon after finishing many years of architectural studies, he experienced serious mental illness that disrupted his plans of working as an architect. Carl has now been ill for more than ten years. He leads a busy life, engaging in activities at the community mental health centre and in a religious community. Through our analysis, we noticed that Carl often makes use of his architectural knowledge and abilities in present everyday activities, such as in the art-group. “*The architect in me is visible in my pictures*”, he told the first author. The first author also observed that others often talk about, make use of, and praise Carl for his architectural knowledge, in our understanding thus assisting Carl in enacting a role of being an architect. We understand that this role helps connect Carl’s past, present and future activities and that it is a potential plot of narrative meaning that several persons share and enact through activities and interplays at the centre. However, presenting new challenges and perhaps possibility to recover further, Carl imagines working in the future:

During one of our meetings, one of the participants in the art group said to Carl; “I heard that you registered an individual enterprise recently. What does that entail?” Carl confirmed that he had contacted the employment office seeking help to start an enterprise. He was hoping to become engaged in some architectural work. However, he did not quite know how to proceed with this, and said that he would keep receiving support from the employment office.

We found Carl’s efforts to start an enterprise quite fascinating. In most of his everyday activities, Carl sought the initiative and support of others, often within mental health arenas. Now he made his own initiatives and sought support elsewhere, trying out possibilities of acquiring work by contacting the employment office and starting an enterprise. We suggest that although the activities and interplays at the centre contribute to Carl’s plot of being an architect, they are insufficient for trying out possibilities of finding work. Although the visitors and staff there show their interest and support, they do not engage in work-related activities together with him. Neither do they posit knowledge about architectural work, nor strategic positions within the work market that could contribute to Carl finding work. Acknowledging this, Carl contacts the employment office for support.

However, Carl’s efforts to engage in architectural work involved fragile and uncertain dimensions:

The next time we met, I was curious to hear more about Carl’s search for work and asked how things had come about with his enterprise. He smiled and said that not much had happened yet. However, he did have a computer set up with the right architectural programs, and was planning to join an architectural competition, if he could find a work assignment he was interested in doing … I said; ‘oh, it sounds like you have most things in place’. Carl seemed hesitant, and replied ‘yes, but I might have to set up a home office first … ’

These comments of waiting for the right architectural competition, and having to set up an office first, puzzled us. After having started an enterprise together with the employment office, he now seemed left on his own and hesitant to get started. We suggest that Carl lacks opportunities of engaging in activities and places that provide interplays with persons who take part in a collective process of imagining, practicing and negotiating possibilities of work together with him. We propose that, similar to Brad, such interplays are crucial for Carl to continue trying out new possibilities. Therefore, work currently seems unattainable for him, causing him to hesitate and leaving a loose end in Carl’s plot. Thus, Carl’s process of narrative meaning-making and recovery is still ongoing and in suspense, leaving us with an open ending.

As with Brad, these findings show how everyday activities, places and persons are crucial to narrative meaning-making. Specifically, Carl seems to need activities and interplays through which he can test, practice and negotiate possibilities of finding work. This suggests that in each unique process of narrative meaning-making, some activities, places and persons are particularly significant as contributors.

Next, we attend to Mary and our analysis of how she is trying out several possibilities for narrative meaning-making, underlining how recovery is ambiguous and open-ended.

### Working out uncertainties—trying out a plot guiding her in different directions

Mary is a woman in her mid-40 s. As a young adult, she moved to the city to study and then started working as an office assistant. However, after some time Mary experienced mental health problems. She could not manage work anymore and moved back to her hometown to be closer to her family. Currently, Mary lives with her husband. Her parents live nearby, together with her younger brother who has a severe and chronic illness and needs a lot of care.

Throughout her meetings with the first author, Mary often shared her thoughts and wishes about becoming more active and contributing to the society and people around her. Through our analysis, we came to understand that she is currently trying out several possibilities of accomplishing this and that this wish has guided many of her past and current everyday activities, as well as her images for the future:

While working out at the gym, Mary told me that she would eventually like to acquire regular, paid work. She said, “It is kind of a demand you know, that one should work and make oneself useful”. Mary underlined that the money is not that important to her, and further explained, “I try to build trust in the job market through doing volunteer work. However, volunteer work does not demand anything from me, I miss having responsibility. My hope for the future is to acquire paid work within health services”.

Drawing on her experiences of volunteer and paid work, Mary currently imagines possibilities of working again in the future. However, throughout her meetings with the first author, Mary expressed her thoughts and doubts about how to tie together her visions of working with her past and present experiences of mental illness. What would she be able to do? How would working again affect her mental health? Who should she make contact with? Nonetheless, Mary showed and told about engaging in activities that may lead her to become part of the workforce. She was reading literature about health, taking on assignments of both paid, volunteer and charity work, and contacting possible employers. Additionally, Mary mentioned having to regain trust from the job market, suggesting that being let back into the workforce may necessitate negotiations with others. Exemplifying such negotiations, Mary told the first author about her experiences of working in restaurants:

While continuing our workout, Mary told me that she had tried working in restaurants several times. However, she had received feedback on her strengths and limitations from her work leaders and had concluded that working in restaurants was not for her. “I work too slowly”, she said.

Hence, working in restaurants and receiving concrete response from others has caused Mary to deliberate what she is able to do. She seems to agree with these work leaders, admitting that she works too slowly. Accordingly, she adjusts her images for the future, concluding that working in restaurants is not for her. However, despite having to do some trying and failing, negotiations and adjustments, Mary continues her activities of contacting possible employers and taking on volunteer and charity work. Hence, we understand that being an active and contributing person is very important for Mary and that through both thoughts, activities and communication with others concerning work, she is currently trying this out as a plot for narrative meaning-making.

Although Mary seemed very intent on working in the future, she also expressed a strong wish to focus on family matters. Her parents were getting older and would eventually need help taking care of her brother. Mary told the first author that she pondered a lot about whether she should prioritize work or caring for her brother in the future or if it is possible to combine these activities. Both taking care of her brother and acquiring work are future images that we understand may create narrative meaning for Mary, by building on her experiences of work and family life and providing possibilities of being an active and contributing person. Thus, we associate her process of narrative meaning-making with tracing a thematic thread that is frizzled, and which guides her in several possible directions. Mary does not know which strands will lead way further along the thread, and which strands may lead to a loose end. Thus, Mary seems to linger in a process of imagining future possibilities, trying them out through activities and interactions, but then withdrawing again, not yet knowing how to create a working narrative.

Similar to both Brad and Carl, Mary’s ongoing process of narrative meaning-making requires interplay and negotiations with significant persons and places, such as workplaces and employers. Further, Mary imagines several, and somewhat competing, everyday activities that could help create meaning, not knowing which activities will eventually connect her experiences into a coherent narrative. This underlines how ongoing processes of recovery are ambiguous and open-ended.

Lastly, we will attend to Sandra, and our analysis of how her images of what is important to her, drove and guided her everyday activities and contributed to a process of recovery.

### From anxious passivity to user representative—recovery driven by the plot of caring for others

Sandra is a woman in her 40 s, whom the first author met with several times at one of the community mental health centres in the city. In one meeting, the first author commented to Sandra that she stood out as a resourceful and active person at the centre. Sandra confirmed this, but added that it had not always been like this and then told the story about how she became active at the centre:

I have experienced anxiety my whole life. My boyfriend of many years, Tim, thought it would be good for me to go the community mental health center. However, I refused, as I did not dare to go. One day as we were going shopping, Tim told me that he had to run an errand at the center, and that I would have to wait for him there. I waited in in the salon, where I sat quietly, looking down at the floor, listening to the conversations going on around me. As I sat there, I heard people talk about their illnesses, use of medications and their side effects, as well as experiences of hospitalization. Listening to these conversations, I thought about how important it is for me to be able to take care of my kids. In fear of losing this ability, I decided never to become so severely ill that I would have to go through such experiences. Consequently, I suddenly got up from the sofa, walked decidedly into the kitchen, and asked the staff if I could help them. In the years to come, I gradually took on new tasks and responsibilities at the center. Having been active at the center for more than ten years, I now serve as a user representative, organize activities, and support others at the center.

Sandra’s story about how she became active at the centre intrigued us. How was she suddenly able to defy her anxiety and get up from that sofa? Throughout our meetings, Sandra repeatedly underlined how important it is for her to care for her children. She also told several stories about caring for relatives, friends and neighbours both in her past and in present. Hence, caring for others seemed to be an important issue for Sandra. Through analysing Sandra’s story, we understand that she imagined how a worsened mental health could disrupt her ability to take care of her kids and that these upsetting images provided Sandra with motivation to act. Further, based on her previous experiences of caring for others, Sandra had faith in the recovering potential of helping at the centre. This idea of how to get better drove her to get up from the sofa and offer her help to the staff. Thus, at the time of these events, we understand her wish of caring for others as a plot, which drove and guided her activities at the centre.

For Sandra, being able to take care of others was so important, that despite her anxiety she was able to engage in a range of activities to preserve this ability. This shows how powerful individuals’ images of how to create narrative meaning may be as driving forces for activities and recovery. However, Sandra also praised her boyfriend and the staff, underlining how their involvement had been essential for her engagement in activities at the centre. We understand that through their encouragement and actions, they nurtured Sandra’s hope and drive to act, and offered possibilities for Sandra to try out activities through which she could both care for others and get better herself. Thus, several significant persons contributed to the ongoing narrative of Sandra getting well and preserving her caring abilities, and their shared activities at the centre contributed to create meaning and support her recovery.

## Discussion

Guided by a narrative understanding of recovery (P. E. Deegan, 2002; Mattingly, 1998; Roe & Davidson, 2005), the aim of this study was to explore how mental health recovery unfolds through individuals’ engagement in everyday activities. Through our narrative analysis we gained in-depth, processual and contextual knowledge about four unique processes of recovery. Our findings show how both Brad, Carl, Mary and Sandra use their past and present experiences from everyday activities as resources to imagine and try out plots that may support narrative meaning and thereby movement in the process of recovery. Our findings render recovery as ambiguous and open-ended processes of narrative meaning-making, enacted through everyday activities that involve interactions with others, adding to similar findings in other studies (Lindström et al., 2013; Mattingly, 1998; Ulfseth et al., 2015, 2016). Emerging from our analysis, we would like to explore further how we may understand processes of narrative meaning-making in recovery as collective, as well as discuss possible implications for practice based on our findings.

Our findings show how everyday activities put individuals in touch with places and persons. Further, they show how through doing activities together, several individuals share ideas and initiatives, give response to each other, and thereby negotiate and try out possible plots together. In light of these findings, and supported by Bruner’s (1990) writings about “social meaning readiness”, we suggest that engaging in everyday activities with others involves collective processes of narrative meaning-making. As an example, through the initial actions of her boyfriend, and after offering her help, Sandra came in touch with the community mental health centre and the persons there. For Sandra, persons around her seemed to understand and support her plot of caring for others and therefore provided her with opportunities of engaging in activities and interplays that aligned with this plot. Another example is how Mary, while trying out the plot of being an active and contributing person, engaged in work-related activities that implied interactions and negotiations with both current and possible workplaces and employers. These interplays contributed to adjust and guide her further images and actions, thereby enabling her to continue engaging in a process of narrative meaning-making. Thus, these findings suggest that narratives of recovery are assembled by a myriad of connected contributors and events, including everyday activities, the interactions and contributions of several persons, as well as the places accommodating these activities. Both Duff (2016) and Price-Robertson et al. (2017) underline that inter-personal and contextual conditions are crucial components in mental health recovery, and everyday activities (Doroud et al., 2015), relationships (Tew et al., 2012; Topor et al., 2006) and places (Duff, 2012; Myers, 2016) have already been documented as important dimensions in recovery. However, based on our findings we conclude that these are not just components but also active and crucial contributors to recovery. Mental health recovery unfolds beyond the individual’s efforts; processes of recovery are unique—but not individual.

Further, our findings underline how there seems to be certain activities, places and persons that are crucial as contributors in each unique process of narrative meaning-making. These findings are also supported by Duff (2012), who concludes that a place that is enabling for one individual, may not be enabling for another. Unfortunately, relevant contributors may not always be available or in agreement on what possibilities to try out, leaving the process of narrative meaning-making complicated or stranded. An example from our findings is how Carl needs to engage in activities that put him in touch with places and persons that are significant for trying out possibilities of acquiring work. However, currently, he seems left on his own without possibilities of such interplays, causing a halt in his process of narrative meaning-making. Another example is how Brad asks the staff at the community mental health centre to engage with him in organizing yoga-classes, but experiences that they do not respond positively to his initiative, leaving his process of trying out this activity stranded. Professionals are encouraged to promote individuals’ drive to act, through inspiring their belief in possibilities of recovery, to imagine recovery narratives, and to have faith in their own abilities to affect their future (P. E. Deegan, 2002). However, our findings of how recovery processes are dependent on the active engagement of several contributors make us wonder: Is it possible to facilitate the collective imagination, hope and enactment of narratives of recovery? Are there efforts professionals could make to locate and inspire several of the crucial contributors in each unique process of recovery?

We do not have any clear answers to these questions. However, based on our findings we understand that facilitating such collective processes demands specific and contextual knowledge about the unique process at hand, implying close collaboration between individuals involved, both service users, professionals and others. Further, it demands creativity: imagining and trying out how new activities, places and persons can contribute to each particular process of meaning-making. In Brad’s case, for example, this collective process seems to have stranded, as the staff at the centre has not responded to his initiatives. Important concerns in this case could be to find out how to facilitate interplays that help Brad proceed. Are there other persons or places he can approach that would engage in trying out yoga with him? Alternatively, are there other activities he could try out, that would engage others at the centre more easily? Similarly, in Carl’s case, what places and persons could take part in trying out possibilities of working? Would it be helpful to contact a person who is an architect, and who could engage in work-related activities with Carl? Would a company be willing to take him in as a trainee? Both Myers (2016) and Duff (2012) similarly suggest that mental health professionals may have a role in helping individuals gain access to, or cultivate, local places and interplays which may contribute to processes of recovery. Myers (2016) also underlines that to acquire the opportunities needed to recover, individuals may have to move beyond professionalized mental health arenas and to other arenas such as religious communities, employment or education settings, or family and peer-networks. In the recent years, new arenas focusing on coproduction of mental health services have emerged, such as recovery colleges (Newman-Taylor et al., 2016) and clubhouses (Chen, 2017; Tanaka & Davidson, 2015). These organizations are run by students/members and professionals together and seek to create meaning and movement in people’s everyday lives through collective activities such as teaching courses and work projects. While carrying out their collective projects clubhouses tailor tasks and activities to their members’ personal pursuits and talents to elicit movement in their process of recovery (Chen, 2017). Further, through their collaboration with other organizations in the community, these arenas offer possibilities of creating relationships between members and persons and arenas outside the clubhouse that are valuable to collective recovery (Crowther et al., 2019). Thus, clubhouses offer both arenas of engaging in collective activities, as well as pursuing personal goals and wishes through specific activities, relationships and arenas relevant to each unique recovery process, and may be very valuable in facilitating collective recovery processes such as the ones we have presented in this article.

## Methodological considerations

In this study, we chose to create data through participant observations, which allows for rich and contextual knowledge by collecting data through several meetings, situations and over time (Fangen, 2004). We assessed writing field notes to be the most suited way to record contextual, action-focused data, and therefore chose not to tape-record the meetings. However, doing activities trigger imagination and associations, allowing for spontaneous conversations relevant to current activities and situations. Therefore, when analysing and interpreting the data we explored the first author’s notes on both what was done and said during the meetings.

In ethnography researchers cannot avoid having an effect on the phenomena we study (Hammersley & Atkinson, 2007), therefore, reflexivity regarding our impact on the data, analysis and interpretations is important. Hammersley and Atkinson (2007) suggest that rather than trying to eliminate the effects of the researcher, we should try to understand and exploit them. The first author created the data together with the participants, and participant–researcher interactions and conversations were analysed by all authors as part of the data-material. On occasion, the first author influenced the focus and richness of the data by inviting conversations relevant to our study aim. The authors’ sought to remain open and curious about the unique situations of the participants both during data creation and analysis. However, theoretical and empirical knowledge, as well as our professional experience as mental health workers and occupational therapists, inspired and informed our analysis and interpretations.

When searching for connections between different parts of the data material with a narrative orientation as we did in our analysis, it is important to remain open to the ambiguity and uncertainty of one’s interpretations (Hammersley & Atkinson, 2007). As our findings show, we explored contradictions in our data material, discovering how unfolding processes of recovery are permeated with both complexity and uncertainty, allowing for many different narrative possibilities and interpretations. We engaged in a systematic analytical process of writing fieldnotes, reading and discussing the field notes in the research group, drawing on theoretical and empirical knowledge, and discussing findings and interpretations in the research group as well as with the participants. We communicate this process thoroughly in this article, making it as transparent as possible, and argue that our findings and interpretations may be recognizable and of value to others. However, we underline that our interpretations are only some of many possible.

This is a study with only four participants, providing in-depth knowledge related to its aim. We suggest more research is needed to nuance and deepen further our processual and contextual knowledge about mental health recovery.

## Conclusion

Building on narrative theory we have argued that individuals create meaning through their activities and that such meaning-making processes offer possibilities of transformation and recovery. We therefore chose to focus on everyday activities in this study, and by use of a narrative-in-action approach, we have created processual and contextual knowledge showing how doing everyday activities opens possibilities of creating meaning and recovery together with others. This study is important as it answers to a reported lack of processual and contextual knowledge about how mental health recovery is interrelated with doing everyday activities (Doroud et al., 2015; Duff, 2016; Ellison et al., 2018; Price-Robertson et al., 2017; Topor et al., 2011).

To conclude, our analysis, interpretations and discussion have shown how recovery unfolds as unique, open-ended and collective processes of trying out plots that may contribute to narratives of recovery. In line with this conclusion, we suggest that a focus on person-centred services (Davidson et al., 2017; Reed et al., 2017) should be supplied with activity-based and coproduction-oriented services when supporting recovery.

We suggest that furthering our understanding of mental health recovery requires methods such as the one applied here, as it accommodates the complexity and uncertainty of these processes. An interesting focus for future research would be to continue exploring mental health recovery as collective processes, seeking more knowledge about how we can facilitate such processes.
